# Inequality in access to COVID-19 vaccines: an annual experience in Verona (Italy)

**DOI:** 10.1093/eurpub/ckac131.115

**Published:** 2022-10-25

**Authors:** A Sartorello, E Paiola, F Moretti, L Accordini, C Postiglione, S Tardivo, R Benoni

**Affiliations:** 1 Department of Diagnostics and Public Health, University of Verona, Verona, Italy; 2 Prevention Department and Public Health, AULSS 9 Scaligera, Verona, Italy

## Abstract

COVID-19 vaccination campaigns involved massive resources worldwide. However, the disparity in vaccine accessibility is a global issue. The study evaluated whether birthplace is a barrier to healthcare access in a high-income country (HIC). The retrospective cohort study included fully vaccinated adults in the Verona district between 27/12/2020 and 31/12/2021. In Italy, the vaccination was opened at different times according to the risk category. Two multiple linear regression models explored the relationship between (1) days before getting the first shot (IV) and (2) the distance between the municipality of residence and the vaccination point, and age, sex, and Income Group (IG, as defined by the World Bank). Distance (km) was estimated with Q-GIS. Results are reported as Marginal Effect at the Mean (MEM) with a confidence interval of 0.95. 500,001 first doses were included, with a mean age of 47 years (SD = 21) and a mean IV of 47.5 days. 6% of the sample was UpperMiddle (UMIC), 6% Lower-Middle (LMIC), and 0.3% Low-Income Countries (LIC). The mean age was higher for HIC (p < 0.05). Male outnumbered females in LMIC (61%) and LIC (69%), but not in HIC and UMIC (p < 0.001). LMIC and LIC were vaccinated at local facilities (5.8%) and pharmacies (4.2%) more than other groups (3%) and at hub centers less (p < 0.05). The IV was lower for subjects from HIC (p < 0.05) with a MEM of 24 [22; 26] for LIC, 21 [21; 22] for LMIC and 27 [26; 27] for UMIC. Men from UMIC (9 [4; 14]), LMIC (7 [6; 8]) and LIC (4 [3; 5]) had a higher IV than women. All variables being equal, IV decreased with age (MEM -0.48 [-0.49; -0.47]). Distance was shorter for LMIC and LIC than for HIC (p < 0.05). The MEM on the distance of the Income group was -2.8 [-3.5; -2.2] for LIC and -2.0 [-2.1; -1.8] for LMIC (p < 0.05). The Income Group of one’s birth country is a barrier to vaccine accessibility in Italy, a HIC. Hence, we address public health workers to improve access to vaccination in community settings to narrow this gap.

**Key messages:**

• Birthplace Income Group could be linked to vaccine accessibility in High Income Countries.

• Public Health stakeholders should consider community and social barriers to healthcare access when planning health interventions.

